# Patient Anxiety About Immediately Released Laboratory Test Results

**DOI:** 10.1001/jamanetworkopen.2026.40246

**Published:** 2026-10-20

**Authors:** Uday Suresh, Bryan D. Steitz, Brian Carlson, Jaqui Dixon, Brent N. Rexer, Megan E. Salwei, S. Trent Rosenbloom, Jessica S. Ancker

**Affiliations:** 1 Department of Biomedical Informatics, Vanderbilt University Medical Center, Nashville, Tennessee; 2 Vanderbilt University Medical Center, Nashville, Tennessee

## Abstract

This survey study examines patients’ anxiety while waiting for laboratory test results, changes in anxiety after viewing results, and intention for viewing future results.

## Introduction

Since the 21st Century Cures Act Information Blocking Rule took effect in April 2021, US medical centers have generally released test results immediately through patient portals rather than withholding them for clinician review.^1,2^ Most patients value immediate access even when results are abnormal,^2^ but patients receiving results (especially abnormal or sensitive ones) without a clinician present may experience worry or anxiety.^3,4^ Anxious patients risk making poor decisions^5,6^ and may send more messages and schedule more appointments.^4,7^ Our objective was to measure anxiety, anxiety change, and preferences for viewing future results, seeking to improve self-report validity by surveying patients immediately after an encounter, confirming in electronic health record metadata that they had viewed results, and extracting objective counts and types of results.

## Methods

This cross-sectional survey at Vanderbilt University Medical Center in Nashville, Tennessee, was conducted by adding questions to a routine patient experience questionnaire (Press Ganey Outpatient Medical Practice Survey) sent electronically to all patients immediately after encounters. From September through November 2025, patients who reported having viewed test results in the past month received study questions about anxiety or worry while waiting for test results, change in anxiety or worry after viewing results, and intention for viewing future results, using ad hoc questions (eFigure 1 in Supplement 1). Only patients who were confirmed to have viewed results in the previous 45 days were included (eFigure 2 in Supplement 1). We extracted counts and characteristics of their test results, including whether the test fell into a category considered sensitive (eTable in Supplement 1) and whether the result was abnormal. The Vanderbilt University Medical Center institutional review board determined the study exempt because data were deidentified. This study follows STROBE and AAPOR reporting guidelines.

Multivariate logistic regressions are reported, with results expressed as odds ratios (ORs) with 95% CIs. All tests were 2-sided at α = .05, using R statistical software version 4.5.0 (R Foundation for Statistical Computing). Additional details are shown in the eMethods in Supplement 1.

## Results

From 234 354 distributed surveys, 46 000 (19.6%) were completed. Of these, 13 898 were from unique patients who met all inclusion criteria. The median (IQR) age was 67 (58-74) years, 7988 patients (57.5%) were female, and 5398 (38.8%) had commercial insurance (Table). Two-thirds had received at least 1 abnormal test result in the previous 45 days, and 12 397 (89.2%) received at least 1 test deemed sensitive by our medical center.

**Table.** Demographics, Clinical Characteristics, and Tests for Included and Excluded Patients

Characteristic	Survey respondents, No. (%)	
	With confirmed result review (n = 13 898)	With no result review (n = 11 854)
Age, median (IQR), y	67 (58-74)	67 (57-75)
Sex		
Female	7988 (57.5)	7309 (61.7)
Male	5910 (42.5)	4545 (38.3)
Race^a^		
Black	1094 (7.9)	876 (7.4)
White	11 961 (86.1)	10 003 (84.4)
Other	842 (6.1)	973 (8.2)
Language		
English	13 773 (99.1)	11 745 (99.1)
Other	125 (0.9)	109 (0.9)
Insurance		
Commercial	5398 (38.8)	4652 (39.2)
Medicaid	127 (0.9)	117 (1.0)
Medicare	4923 (35.4)	4031 (34.0)
Other	3450 (24.8)	3054 (25.8)
Elixhauser comorbidity count, median (IQR)	0 (0-2)	0 (0-1)
No. of tests received, median (IQR)	6 (3-10)	0 (0-1)
Received abnormal test result	9436 (67.9)	1385 (11.7)
Portal account age, median (IQR), y	5 (2-8)	4 (1-7)

^a^ Race and other variables were extracted from electronic health record data. Race was categorized as White, Black, or other (including American Indian or Alaska Native, Asian, Native Hawaiian or Pacific Islander, multiracial, unknown, and declined). These are reported to assist readers in assessing generalizability of the sample, and because demographics have previously been reported to be associated with anxiety disorders, health anxiety, and portal use.

Of the respondents, 3881 (28.0%) experienced reported feeling somewhat, very, or extremely worried or anxious while waiting to view their test results (Figure). Feeling extremely worried at baseline was associated with female sex (OR, 1.39; 95% CI, 1.12-1.74), higher comorbidity burden (OR per Elixhauser condition, 1.09; 95% CI, 1.04-1.14), and being in the 35- to 49-year age category rather than any other (eg, 35-49 years vs <35 years, OR, 1.98; 95% CI, 1.20-3.39), but not with whether any of their tests fell into the sensitive category.

**Figure.**
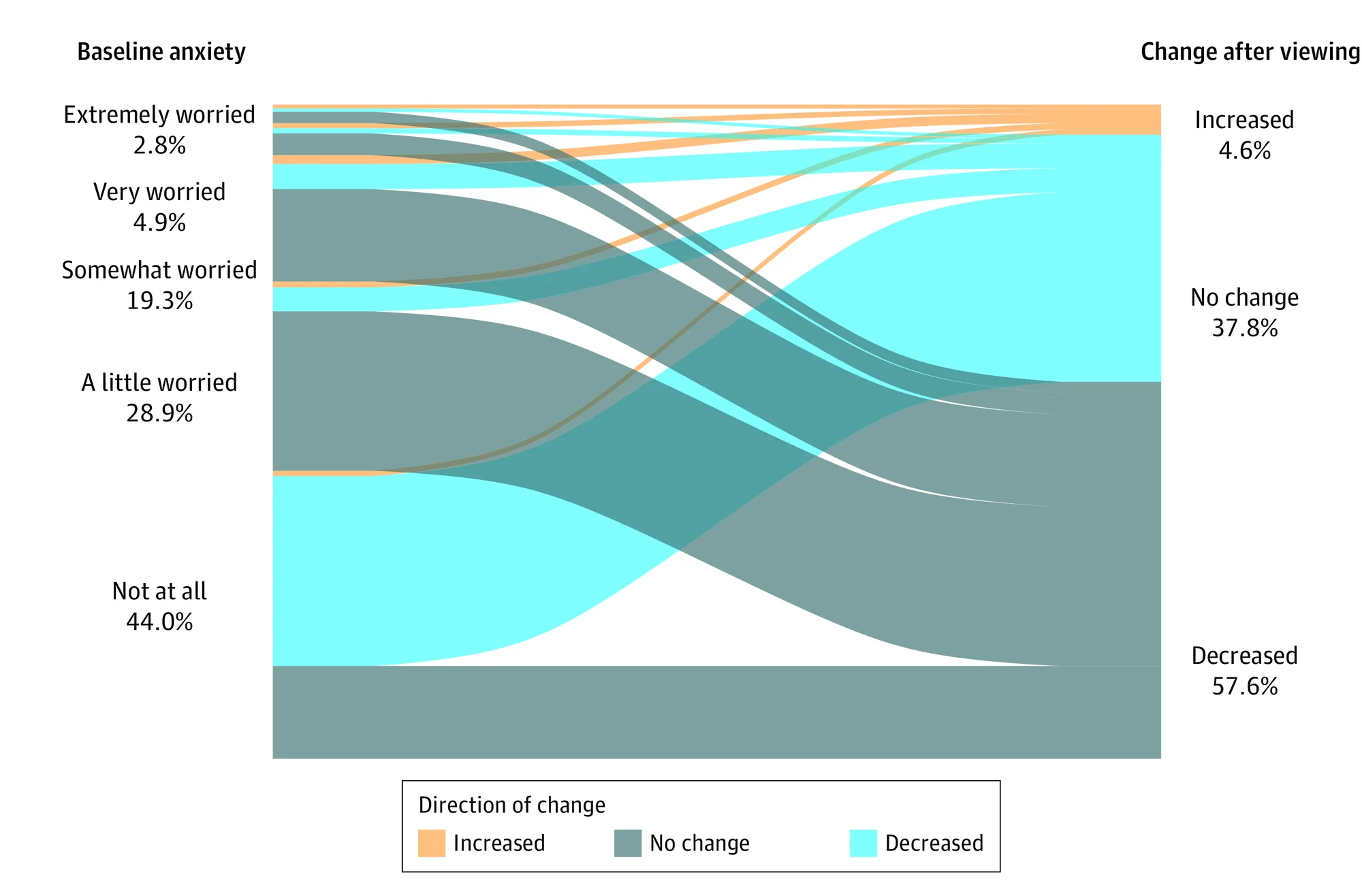
Sankey Diagram of Baseline Anxiety and Change After Viewing Results Most patients, including those who experienced worry at baseline, experienced a reduction in anxiety after viewing results. Figure excludes 264 patients (1.9%) who did not answer the anxiety change question.

Most respondents felt less anxious or experienced no change after viewing their results, but 4.6% (629 respondents) reported feeling a little more or much more worried or anxious (Figure). Experiencing an increase in anxiety after viewing the test result was associated with having had higher (vs lower) anxiety at baseline (OR, 2.02; 95% CI, 1.89-2.16). Increases in anxiety were not associated with receiving an abnormal test result (OR, 1.17; 95% CI, 0.96-1.43) or with undergoing a sensitive test (OR, 1.13; 95% CI, 0.94-1.36). However, increased anxiety was associated with 1 category of sensitive test, those that were potentially cancer diagnostic (OR, 1.55; 95% CI, 1.13-2.10). Older age was negatively associated with experiencing increased anxiety (≥80 vs <35 years, OR, 0.40; 95% CI, 0.23-0.68).

Overall, 12 095 patients (88.3%) planned to review future results immediately, 766 (5.6%) planned to wait to speak to a clinician, and 840 (6.1%) were uncertain. Intention to wait for a clinician in the future was associated with higher (vs lower) baseline anxiety (OR, 1.13; 95% CI, 1.05-1.21) and with experiencing increased anxiety after viewing (OR, 2.16; 95% CI, 1.63-2.82). However, even among patients reporting increased anxiety, 497 (79.5%) planned to view future results immediately.

## Discussion

In this survey study, a minority of patients reported anxiety while awaiting test results, most experienced decreased anxiety after viewing results, and most intended to view future results immediately rather than wait for a physician. Receiving abnormal test results in general was not associated with experiencing an increase in anxiety, although receiving a cancer-diagnostic result was. Patients with high baseline anxiety were more likely to experience anxiety increases after viewing results.

Limitations include the use of nonvalidated measures for anxiety, assessment of baseline anxiety after patients had already viewed their results, self-selection to respond to the survey, and a sample drawn from a single academic medical center with a largely insured population. All forms of public opinion research are subject to unmeasured error.

Patients with anxiety around test results should be identified and supported before and during results release. Balancing transparency with individualized support for vulnerable patients is a core challenge for optimizing patient portal experiences in the era of immediate result access.

## Notes

Supplement 1. eMethods. eFigure 1. Questions added to Press Ganey Outpatient Medical Practice Survey (PGOMPS) survey instrument, sent to patients after their encounter eFigure 2. CONSORT-style flow diagram eTable. Vanderbilt University Medical Center historical categorization of test results as sensitive or not sensitive, with release delays prior to 21st Century Cures Act

Supplement 2. Data Sharing Statement
