# Supplementary material for: Patient Anxiety About Immediately Released Laboratory Test Results
Source: JAMA Netw Open. 2026 Oct 20;9(10):e2640246. doi: 10.1001/jamanetworkopen.2026.40246 (PMC13684481; doi:10.1001/jamanetworkopen.2026.40246)
Supplement: Supplementary file 1 [file jamanetwopen-e2640246-s001.pdf]

Supplemental Online Content

Suresh U, Steitz BD, Carlson B, et al. Patient anxiety about immediately released laboratory test results. *JAMA Netw Open*. 2026;9(10):e2640246. doi:10.1001/jamanetworkopen.2026.40246

eMethods.
eFigure 1. Questions added to Press Ganey Outpatient Medical Practice Survey (PGOMPS) survey instrument, sent to patients after their encounterFlow of information through the different phases of the review
eFigure 2. CONSORT-style flow diagram
eTable. Vanderbilt University Medical Center historical categorization of test results as sensitive or not sensitive, with release delays prior to 21st Century Cures Act

This supplemental material has been provided by the authors to give readers additional information about their work.

© 2026 Suresh U et al. *JAMA Network Open*.

eMethods

Study Design and Setting
We conducted a cross-sectional survey of patients at Vanderbilt University Medical Center (VUMC), a large urban academic medical center in Nashville, TN. We added survey questions to a routine patient experience questionnaire (Press Ganey Outpatient Medical Practice Survey; PGOMPS) that was sent electronically immediately after each encounter. Our questions were presented to patients who answered that they had viewed test results in the patient portal, and were deployed from September 03, 2025, to November 11, 2025, spanning 69 days. The study was approved by the VUMC Institutional Review Board and determined to be exempt because we were using deidentified survey data.

Inclusion criteria
Eligible patients had an ambulatory healthcare encounter at VUMC between September and November 2025, had an active portal account, were 18 or older, responded to the survey about the encounter, reported (in the survey) viewing test results in the portal within the past month, and were confirmed in the portal data to have viewed a test result. Because of the ambiguity about how "in the last month" could be interpreted, we included patients who had viewed a test result at any time in the 45 days before they answered the questionnaire. When patients had multiple encounters and completed more than one questionnaire, we selected the patient's first encounter for analysis.

Survey Instrument
We supplemented the existing PGOMPS with a question about whether the respondent had viewed a test result in the last month. For those who answered yes, we asked three additional questions about their feelings and intentions about "the most important or significant result" they had viewed (Supplemental Figure 1). The first asked about their anxiety before the test result arrived (on a 5-point Likert scale from 1=not at all worried to 5=extremely worried). The second asked about change in anxiety after viewing the result (5-point scale from 1=much less worried to 5=much more worried). The third asked about viewing intentions for future test results, with three response options: "review it immediately," "wait until you can speak with your doctor about it," or "I don't know." The patient experience survey was administered electronically with patients accessing the survey through email or text message immediately after a closed encounter.

Measures
We developed the anxiety and anxiety-change questions de novo after determining that no existing validated anxiety-related questionnaire specifically focused on anxiety related to medical test result release.

From the EHR and usage logs, we extracted respondent demographics, clinical characteristics (inpatient and outpatient diagnoses over 2 years to compute Elixhauser Comorbidity Index, presence of anxiety diagnosis), patient portal use information (duration of account, and whether patients had opted in to receive electronic notifications about new test results), and features of the lab tests delivered to the portal over the 45 days before the survey was completed. These included the broad clinical category (e.g., cancer diagnostic test, genetic test, etc.), whether the test produced an abnormal finding, and whether the test fell into a category considered by VUMC to be sensitive (i.e., a test that prior to 2021 would have been released only after a 7 or more day delay). Examples of these so-called sensitive previously delayed tests include pathology, HIV tests, anatomic pathology reports, and genetic tests. We created a new variable, had a cancer-diagnostic test, on the basis of whether the patient had undergone one of any tests on an expert-derived list of pathology tests. Because our focus was on results review, we did not define new cancer diagnosis through the presence of new cancer-related ICD codes in the record; the addition of a new ICD diagnosis to the patient record might or might not occur at the same time as the delivery of new results to the portal. For analysis, we aggregated tests delivered to the patient to determine whether the patient had received any abnormal results, results from a sensitive test, or cancer-diagnostic tests during the 45 days before completing the questionnaire.

© 2026 Suresh U et al. *JAMA Network Open*.

Statistical Analysis

For descriptive statistics, continuous variables were summarized as median and interquartile range (IQR) and categorical variables were summarized as frequencies and percentages. Sample sizes varied between individual analyses as differing numbers of patients answered each of the questions and descriptive statistics used all available data for each variable. Age was categorized as 18–34, 35–49, 50–64, 65–79, and ≥80 years. This was a non-probability sample, and no weights were applied. Multivariable logistic regression was used to model predictors of increased anxiety after viewing. All predictors were incorporated into multivariable models for the 3 primary outcomes. We set α to 0.05 for significance testing. All analyses were performed using R version 4.5.0 (R Foundation for Statistical Computing).

© 2026 Suresh U et al. *JAMA Network Open*.

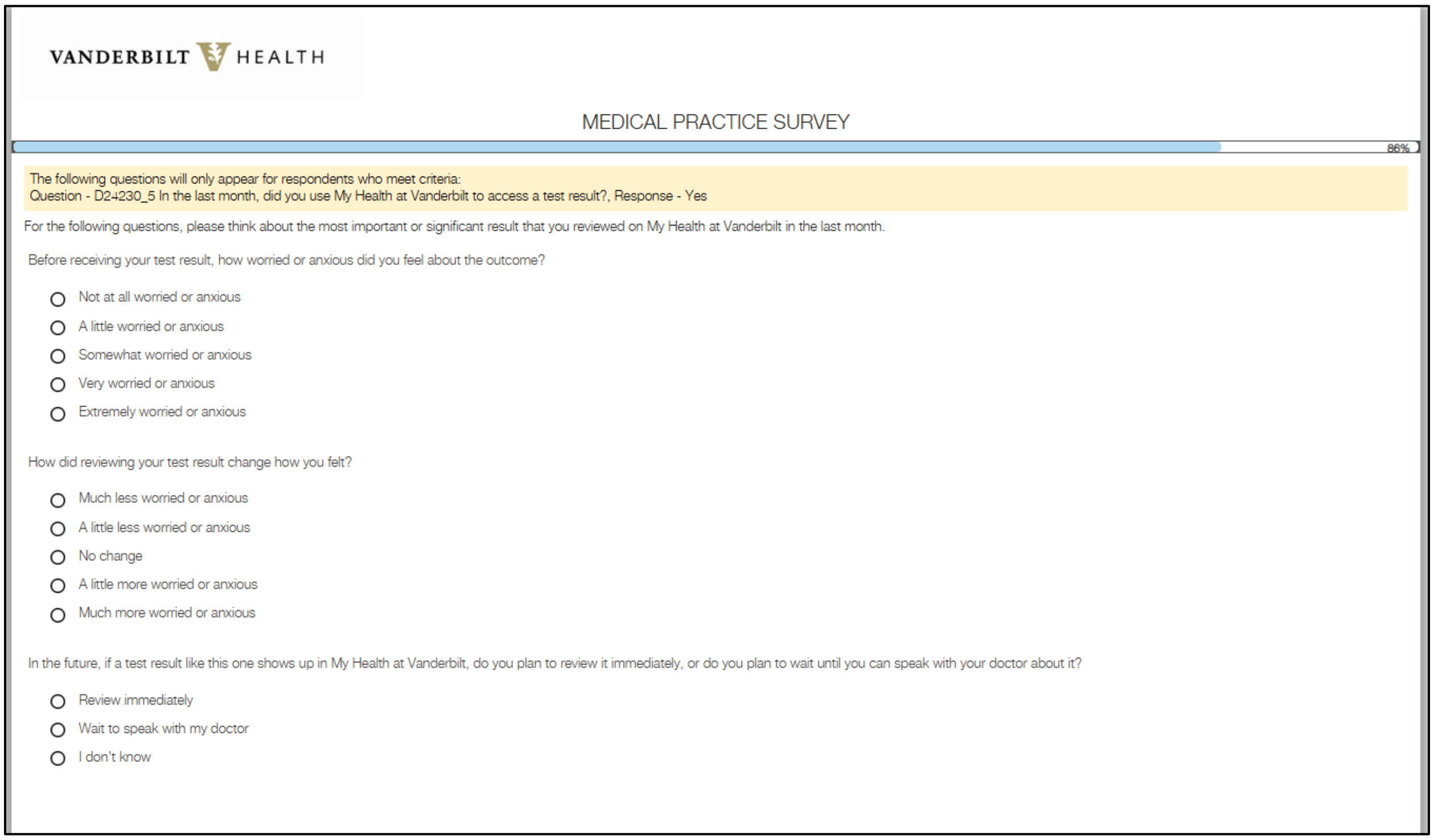

VANDERBILT HEALTH

MEDICAL PRACTICE SURVEY

86%

The following questions will only appear for respondents who meet criteria:
Question - D24230_5 In the last month, did you use My Health at Vanderbilt to access a test result?, Response - Yes

For the following questions, please think about the most important or significant result that you reviewed on My Health at Vanderbilt in the last month.

Before receiving your test result, how worried or anxious did you feel about the outcome?

- Not at all worried or anxious
- A little worried or anxious
- Somewhat worried or anxious
- Very worried or anxious
- Extremely worried or anxious

How did reviewing your test result change how you felt?

- Much less worried or anxious
- A little less worried or anxious
- No change
- A little more worried or anxious
- Much more worried or anxious

In the future, if a test result like this one shows up in My Health at Vanderbilt, do you plan to review it immediately, or do you plan to wait until you can speak with your doctor about it?

- Review immediately
- Wait to speak with my doctor
- I don't know

eFigure 1. Questions added to Press Ganey Outpatient Medical Practice Survey (PGOMPS) survey instrument, sent to patients after their encounter. The survey automatically implemented a 90-day washout period per patient-provider pair, but patients seen by multiple providers within 90 days were able to receive multiple surveys. When patients submitted more than one survey, we used only the first.

© 2026 Suresh U et al. *JAMA Network Open*.

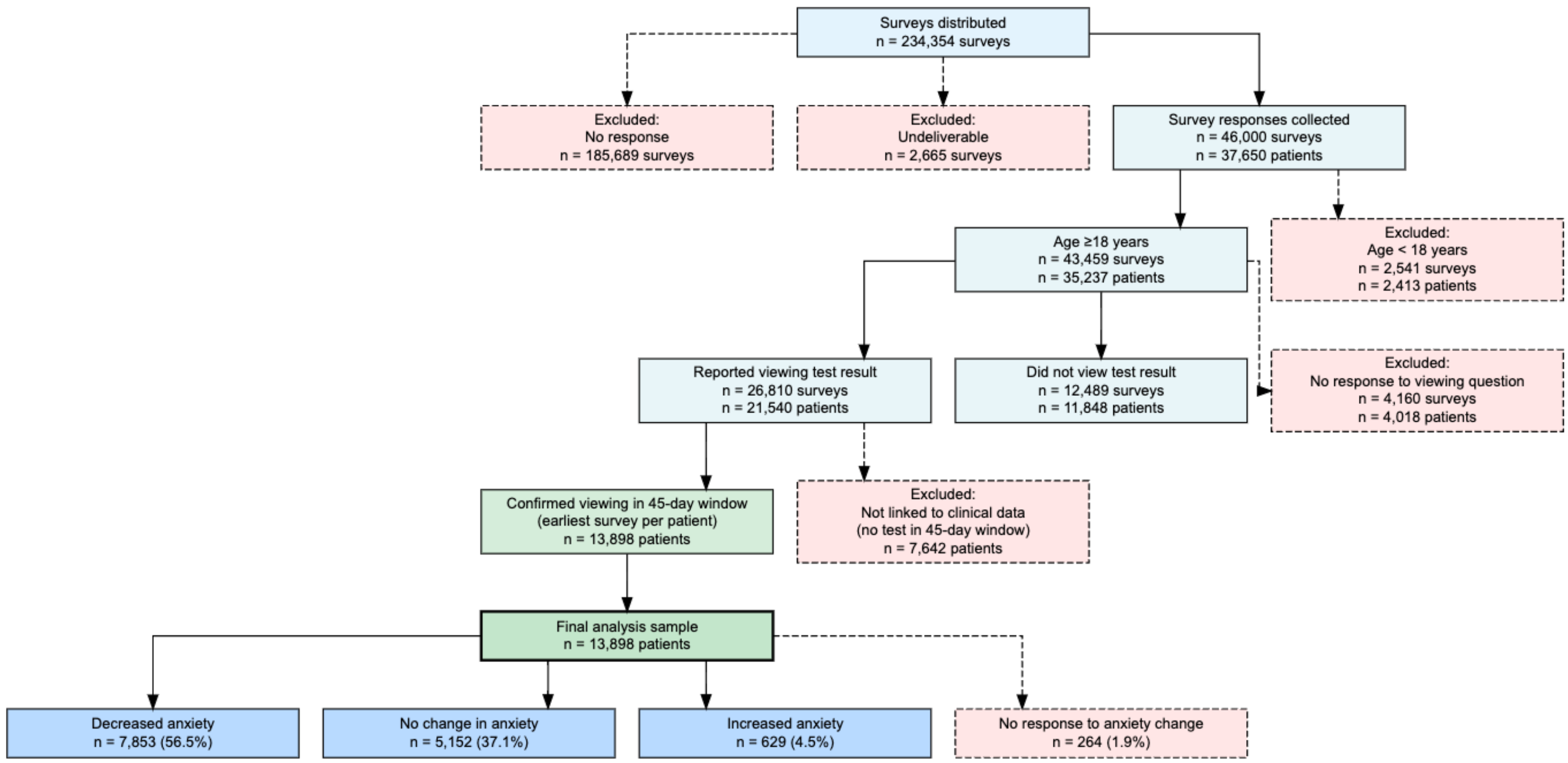

eFigure 2. CONSORT-style flow diagram.

© 2026 Suresh U et al. *JAMA Network Open*.

eTable. Vanderbilt University Medical Center historical categorization of test results as sensitive or not sensitive, with release delays prior to 21st Century Cures Act

Result Category	Sensitivity	Release time prior to 21st Century Cures Act	Representative Procedures
Routine laboratory tests	Not sensitive	Same day	Basic metabolic panel, CMP, lipid panel, renal panel, HbA1c, CBC
Standard laboratory tests	Not sensitive	1 day	CEA, cortisol, FSH, LH, testosterone, aldosterone
Other tests	Not sensitive	3 days	ECG/EKG, CT simulation, EMG, retinal screening
Sensitive laboratory tests	Sensitive	7 days	Drug levels (carbamazepine, digoxin, phenobarbital, phenytoin), HIV, STI panels, hepatitis serologies
Other sensitive tests	Sensitive	7 days	Screening mammogram, digital breast tomosynthesis
Highly sensitive laboratory tests	Highly sensitive	14 days	Pap smear, gynecologic cytology, bone marrow exam, pathology specimens, liver tissue assays
Other highly sensitive tests	Highly sensitive	14 days	Ultrasound-guided biopsy (thyroid, breast), chorionic villus sampling, amniocentesis, core needle biopsy

© 2026 Suresh U et al. *JAMA Network Open*.
