# Supplementary material for: Patient Anxiety About Immediately Released Laboratory Test Results
Source: JAMA Netw Open. 2026 Oct 20;9(10):e2640246. doi: 10.1001/jamanetworkopen.2026.40246 (PMC13684481; doi:10.1001/jamanetworkopen.2026.40246)
Supplement: Supplementary file 2 [file jamanetwopen-e2640246-s002.pdf]

Data Sharing Statement

Suresh. Patient Anxiety About Immediately Released Laboratory Test Results. *JAMA Netw Open*. Published October 26, 2026. doi:10.1001/jamanetworkopen.2026.40246

Data
Data available: No

Additional Information
Explanation for why data not available: Institutional policy prohibits sharing patient data.
